# The Impact of Yogic Breathing Exercises on Pulmonary Functions in Asymptomatic Smokers

**DOI:** 10.7759/cureus.68466

**Published:** 2024-09-02

**Authors:** Pragyashaa Chaudhary, Ketaki Poorey, Nimarpreet Kaur, Pushpa Lamba, Harminder Kaur, Keerty Mathur

**Affiliations:** 1 Physiology, Faculty of Medicine and Health Sciences, Shree Guru Gobind Singh Tricentenary (SGT) University, Gurugram, IND; 2 Physiology, National Institute of Medical Sciences and Research, National Institute of Medical Sciences (NIMS) University, Jaipur, IND; 3 Physiotherapy, Dolphin (PG) Institute of Biomedical and Natural Sciences, Dehradun, IND

**Keywords:** 40 mmhg, om chanting, nicotine dependence, anb, pranayam, ftnd, bhte, bhti, asymptomatic smokers

## Abstract

Introduction

Smoking is a serious public health problem that leads to chronic obstructive pulmonary disease (COPD). Its harmful effects are more significantly seen in the respiratory system. Approximately 30% of smokers experience compromised lung functions. However, asymptomatic smokers exhibit alteration in lung morphology.

This study will give us a better understanding of the respiratory damages that may occur in asymptomatic smokers and if we can delay or prevent the same by the simple practice of Pranayama and Om chanting.

Materials and methods

An interventional study was conducted in the Department of Physiology at the Faculty of Medicine and Health Sciences, Shree Guru Gobind Singh Tricentenary (SGT) University, Gurugram, Haryana, India. The study duration was eight weeks, and a total of 135 subjects, including both male and female participants aged between 18 and 25 years, were included in this study. Baseline recordings of anthropometric parameters, spirometric parameters like forced vital capacity (FVC), forced expiratory volume (FEV) in 1 second (FEV1), FEV1/FVC ratio, forced expiratory flow (FEF) between 25% and 75% of vital capacity (FEF25-75%), peak expiratory flow rate (PEFR), forced inspiratory vital capacity (FIVC), peak inspiratory flow rate (PIFR) and for respiratory endurance - breath holding time at the end of inspiration (BHTi), breath holding time at the end of expiration (BHTe), 40 mmHg were recorded before the start of the study and again after eight weeks of alternate nostril breathing and om chanting performed for 10 minutes (5 minutes for each protocol). We also recorded nicotine dependence score with the help of Fagerstrom Test for Nicotine Dependence (FTND). A comparison of spirometric parameters, respiratory endurance, and FTND was done using the paired t-test.

Result

All spirometric measures and respiratory endurance parameters showed significant increases, with the exception of FVC, FEV1, FEV1/FVC, and FTND, which showed no significant improvement.

Conclusion

Pranayama and Om chanting were found to increase respiratory muscle endurance and support better utilization of the alveolo-bronchial tree, which may help in better oxygenation and delay in onset of the symptoms of COPD.

## Introduction

According to recent reports, approximately 1.2 billion people smoke worldwide, and it is the leading cause of preventable death [1]. Smoking is a significant public health issue that leads to various chronic diseases, such as coronary heart disease and chronic obstructive pulmonary disease (COPD). The adverse effects of COPD are most significantly seen in the respiratory system [1,2]. Given the lung's large functional reserve capacity, smokers may experience a notable decrease in pulmonary function before they experience respiratory symptoms [2]. The decline in the spirometric parameters is swift compared to the non-smoker counterparts and deteriorates silently through the years.

During normal breathing, the body receives oxygen through blood circulation. However, in individuals who smoke, carbon monoxide (CO) is delivered to the body instead of oxygen, producing acute respiratory difficulties such as shortness of breath and coughing. Smoking reduces lung capacity due to its acidic compounds, which gradually damage the lining of the bronchi and bronchioles. This damage leads to inflammation and infection, resulting in symptoms such as coughing, shortness of breath, and chest pain, ultimately leading to chronic bronchitis [1].

The prevalence of COPD is steadily increasing. Around 30% of smokers do not experience chronic symptoms or compromised lung function. However, these "healthy smokers" show alterations in lung shape and symptoms of lung inflammation. It is important to note that smoking always harms the lungs, yet the extent and severity of these alterations vary [3].

This major problem is composed by the fact that even after the awareness and research on harmful effects on the respiratory and cardiovascular system and known major risks of smoking, the rate of smoking continues to rise. This study will give us a better understanding of respiratory damage and whether we can prevent or delay the damage caused by smoking by practicing Pranayama and Om chanting, as very few researchers have focused on asymptomatic smokers.

Both Pranayama and Om chanting are yogic breathing protocols that may affect different systems of the body in a beneficial way, especially the respiratory system. Pranayama is a gentle, yogic breathing technique that causes no stress and improves the autonomic processes of individuals. In the past, it has been added to COPD patients' current therapy with different degrees of success [4].

Pranayama has beneficial and therapeutic effects on the respiratory function of both healthy and diseased individuals. It increases the expansion of the chest wall and makes effective use of the diaphragmatic and abdominal muscles, thereby strengthening the respiratory system. This results in the fullest possible inflation and deflation of the chest and lungs and maximizes the work of the muscles involved [1,5].

This study will give us a better understanding of the respiratory damages that may occur in asymptomatic smokers and if we can delay or prevent the same by the simple practice of pranayama and Om chanting.

## Materials and methods

The study group consisted of 150 asymptomatic tobacco smokers aged between 18 and 25, who were recruited from staff and students at Shree Guru Gobind Singh Tricentenary (SGT) Medical College, Gurgaon. The study was conducted in the Department of Physiology of SGT Medical College, Gurgaon.

The Written informed consent from the students was obtained before the research, and approval was granted by the Institutional Ethics Committee of the Faculty of Medicine and Health Sciences (FMHS), SGT University, Gurgaon (IEC/FMHS/S/15/11/22-58). Then, the subjects were recruited as per the inclusion and exclusion criteria as follows.

Inclusion criteria

Asymptomatic tobacco smokers aged between 18 and 25 years of either sex were included in the study. Smoking history: “Current smoker: An adult who has smoked 100 cigarettes in his or her lifetime and who currently smokes cigarettes daily” (Definition as per National Center for Health Statistics and Global Adult Tobacco Survey India Report 2016-2017).

Exclusion criteria

Medical history of systemic disorders, particularly respiratory tract conditions; and any other allergy conditions; a medical history of consistent medication for any illness; subjects with chest and spine deformities like kyphosis and scoliosis; pregnant, post-partum or lactating females; subjects engaging in any pranayama/yoga/exercise/meditation, etc. before recruitment into the study were excluded.

After obtaining informed consent, anthropometric parameters like age, height and weight were recorded. The baseline data of the spirometry parameters, collected using the digital spirometer RMS Helios 401 (Recorders & Medicare Systems Private Limited, Panchkula, India), like forced vital capacity (FVC), forced expiratory volume in 1 second (FEV1), FEV1/FVC, forced expiratory flow (FEF) between 25% and 75% of vital capacity (FEF25-75%), peak expiratory flow rate (PEFR), forced inspiratory vital capacity (FIVC), peak inspiratory flow rate (PIFR) and for respiratory endurance - breath holding time at the end of inspiration (BHTi), breath Holding Time at the end of expiration (BHTe), 40 mmHg. Fagerstrom test for nicotine dependence (FTND) under research was collected before introducing the intervention. 

Interventions

Subjects practiced the combination of alternate nostril breathing (ANB) and Om chanting before the lunch break around 12- 12:30 pm under supervision, for a duration of 10 minutes (5 mins for each protocol as given below) for the period of six days per week for eight weeks.

Om chanting: Participants were instructed to sit adequately in an easy and steady posture, keeping the head, neck, and trunk erect (Sukhasana), and then take a deep breath. They were then instructed to exhale, and while exhaling, produce the sound "Om" and continue doing so until they could not exhale any further.

For ANB: Participants were instructed to close their right nostril with their right thumb and bring their right elbow to the level of their right shoulder. They were then instructed to slowly inhale and exhale through the left nostril and repeat the procedure through the other nostril.

**Figure 1:**
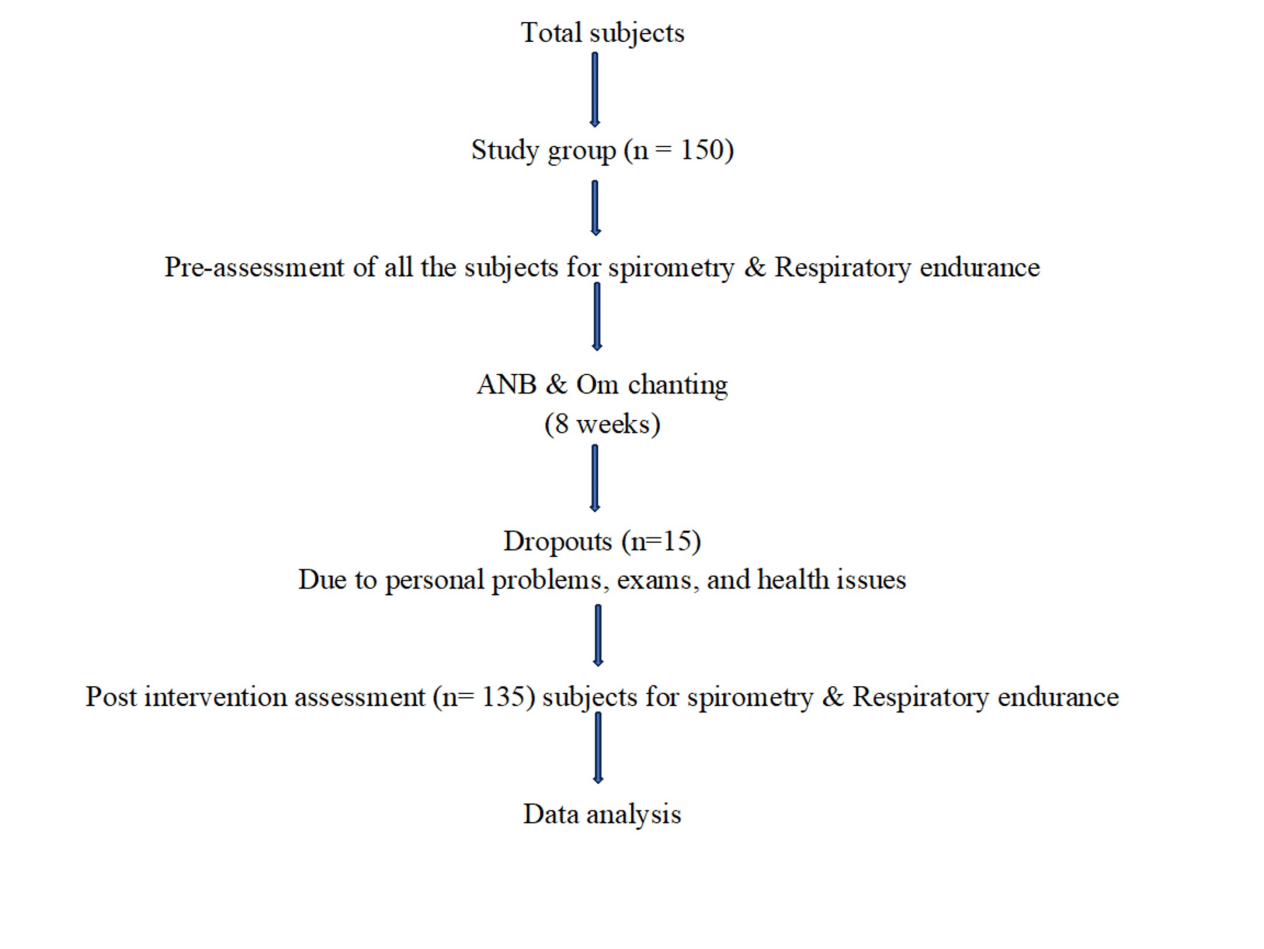
Flow of Events ANB: Alternate nostril breathing

The flowchart in Figure 1 illustrates the progression of the study which started with 150 participants, but due to the dropout of 15 participants, it was finally conducted with 135 participants.

Breath holding time (BHT)

When sitting, the participants were told to hold their breath. They were instructed to hold their breath until they could no longer do so voluntarily, and the duration was recorded using a timer. The breath-holding time was noted at the end of inspiration (BHTi) and at the end of expiration (BHTe).

Respiratory endurance test (40 mm Hg test)

This test utilized a mercury sphygmomanometer. The rubber tubing connecting the mercury reservoir to the BP cuff was detached. The participant was directed to inhale deeply and then close their nostrils, exhaling into the tube until the mercury reached a level of 40 mm Hg. They were then advised to maintain this level for as long as possible. The time was recorded using a stopwatch.

Data analysis

The data was analyzed using statistical analysis of baseline and post-intervention assessments of the total subjects (n = 135). Pre- and post-assessment were analyzed using the paired t-test. P < 0.05 was considered as significant.

## Results

A total of 150 subjects were recruited for the study, and a complete pre- and post-intervention assessment was done for 135 subjects (15 subjects were lost for follow-up assessment) with a mean age of 19.87 years. The other baseline parameters are shown in Table 1.

**Table 1:** Baseline characteristics of the study participants SD: Standard deviation

Parameters	Result
Age in years (mean ± SD)	19.87 ± 1.79
Male (n(%))	74 (54%)
Female (n(%))	63 (46%)
Height in centimeters (mean ± SD)	165 ± 8.6
Weight in kgs (mean ± SD)	62 ± 12
Body Mass Index in kg/m^2 ^(mean ± SD)	22.78 ± 3.87

Table 2 shows that all spirometric parameters, including FEF25-75%, PEFR, FIVC, and PIFR, exhibited significant increases with p values < 0.05. However, FVC, FEV1, (FEV1/FVC) did not show significant improvement.

**Table 2:** Comparison of spirometric parameters before and after eight weeks of Pranayama and Om chanting SD: Standard deviation; FVC: Forced vital capacity; FEV1: Forced expiratory volume in 1 second; FEF25-75: Forced expiratory flow between 25% and 75% of vital capacity; PEFR: Peak expiratory flow rate; FIVC: Forced inspiratory vital capacity; PIFR: Peak inspiratory flow rate. *: p-value<0.0001, p-values are calculated using the paired t-test. Results were considered significant at p <0.05.

Parameters	Before (mean ± SD)	After (mean ± SD)	P value
FVC (L)	2.80 ± 0.56	2.88 ± 0.58	0.07
FEV1 (L)	2.74 ± 0.65	2.80 ± 0.65	0.25
FEV1/FVC (%)	97.84 ± 12.46	97.64 ± 12.65	0.46
FEF25-75% (L/sec)	4.81 ± 1.01	5.03 ± 1.15	0.005
PEFR (L/sec)	6.98 ± 1.52	7.64 ± 1.48	0.0001*
FIVC (L)	2.46 ± 0.62	2.63 ± 0.61	0.0016
PIFR (L/sec)	4.87 ± 1.57	5.48 ± 1.60	0.0001*

After eight weeks of interventions, all respiratory endurance metrics, including 40 mmHg, BHTi, and BHTe, showed significant improvement (p < 0.05), suggesting an increase in respiratory endurance. Meanwhile, FTND showed no improvement after eight weeks, as indicated in Table 3.

**Table 3:** Comparison of respiratory endurance parameters before and after eight weeks of Pranayama and Om chanting SD: Standard deviation; mmHg: Millimeters of mercury; BHTi: Breath holding time at the end of inspiration; BHTe: Breath holding time at the end of expiration; FTND: Fagerstrom test for nicotine dependence *: p-value<0.0001, p-values are calculated using the paired t-test. Results were considered significant at p <0.05.

Parameters	Before (mean ± SD)	After (mean ± SD)	P value
40 mmHg	25.28 ± 9.19	37.88 ± 12.15	0.0001*
BHTi (seconds)	36.15 ± 8.61	50.16 ± 12.94	0.0001*
BHTe (seconds)	24.06 ± 7.01	32.5 ± 8.45	0.0001*
FTND	2.13 ± 1.38	2.53 ± 1.52	0.9

## Discussion

This study is aimed to investigate the efficacy of yogic breathing exercises in healthy smokers. These breathing exercises have been shown to provide significant short and long-term benefits. Our study showed a significant increase in breath holding time (BHT) after inspiration and expiration. Additionally, the 40 mmHg endurance test showed a significant impact on respiratory muscle endurance. We also observed a statistically significant increase in PEFR and PIFR, which again favor the increased effort-dependent parameters. FIVC also increased significantly which may be related to increased muscle endurance, increased central drive, or reduced inflammation with increased nitric oxide (NO). We also noticed a significant increase in FEF25-75%, which favored betterment in the lower airway parameters. Our study also noted an increase in FEV1 and a decrease in FEV1/FVC, but they were not statistically significant. 

The significant increase in respiratory endurance and spirometric parameters is supported by many researches done in the past, in healthy populations and elderly subjects [5,6]. BHT is determined by the initial lung volume, training, and willpower. Pranayama, or intentional control of respiration, can improve respiratory endurance and BHT. According to studies, Anulom Vilom Pranayama influences autonomic functioning by modifying the responsiveness of medullary respiratory centers. It can be concluded that enhanced BHT is the result of several causes, including respiratory muscle strengthening, improved pCO2 clearance, and a lower level of pO2. Both characteristics are obtained by frequent practice of ANB and Om chanting [5-11].

The peak expiratory flow rate (PEFR) primarily reflects the flow of air through the large airways and is dependent on the lung recoil, voluntary effort, and muscular strength of the patient. Additionally, Pranayama, a breathing exercise, trains the respiratory system to achieve complete emptying and filling of the lungs and promotes raising the diaphragm to a higher level. It may also reduce the resistance offered by decreasing the inflammation and increasing the caliber of airways, which may again be mediated by the activation of parasympathetic activity [5,6,9,11-14].

A similar study by Mooventhan and Khode in a healthy population studied various spirometric parameters like FEF25%, FVC, FEV1, FEF25-75%, etc, and their study results stated no significant increase in FVC, and FEF25-75% but significant improvement in FEV1 [15].

In another study conducted by Dullo et al., FEF25-75% increased significantly after four months of exercise as thoracopulmonary compliance increased significantly above the basal level as a result of ANB training [13].

Yogic breathing affects the respiratory system directly as it improves ventilation, which helps the individual to reach maximum utilization of oxygen & maximum expansion of the diaphragm, and this ultimately increases air inhalation, thus improving respiratory muscle strength and flexibility.

Pranayama practice also promotes parasympathetic activity and reduces sympathetic activity. Due to regular practice, the lungs are expanded notably, and there is prolonged inhalation, which stimulates pulmonary stretch receptors. These stretch receptors reduce the tracheo bronchial smooth muscle tone, reducing resistance and increasing airway diameter, culminating in improved flow rates. Since the early involvement in smokers starts with lower airways, the significant improvement in the FEF 25-75% gives us a window to prevent or delay the irreversible pathologic changes in smokers using small and easy exercises [8,16].

Yogic breathing stimulates the vagus nerve and acts on the limbic system, influencing the thalamic nucleus. This results in the activation of the frontal cerebral cortex and the temporoparietal cortex. After practicing pranayama, one may experience lower arousal, improved memory, and balanced emotions. The current researcher concluded that the limbic system might be directly involved in the control mechanism process during pranayama [8,10,17]. In a study by Rosen et al., vinyasa yoga was seen as a positive and potentially helpful technique for quitting smoking. In this study, yoga was an adjunct to cognitive behavioral therapy [18]. In our study, we could not find any significant decrease in the Nicotine dependence measured by the Fagerstorm test after eight weeks of intervention.

Limitations

Our study's limitations include a small sample size, a narrow age range, and a short intervention period. Because our study included only asymptomatic smokers, the findings may not be representative of the symptomatic/unwell population. Studies with COPD patients would have provided a better knowledge of respiratory endurance and nicotine dependence.

## Conclusions

Yogic breathing exercises significantly enhance respiratory parameters in asymptomatic smokers, potentially offering a preventive measure against irreversible pathologic changes in the respiratory system. Better utilization of the alveolo-bronchial tree may help in better oxygenation and delay in symptoms with respect to physiological stressors. In smokers, delaying overt development of COPD.
